# Impact of Antiseizure Medication on Thyroid Function in Children With Epilepsy: A Case-Control Study

**DOI:** 10.7759/cureus.112345

**Published:** 2026-07-09

**Authors:** Nitin Kumar, Priyanka Singh, Vijay K Singh, Suresh N Singh

**Affiliations:** 1 Pediatrics, Baba Raghav Das Medical College, Gorakhpur, IND

**Keywords:** anti-seizure medications, case-control studies, epilepsy, levetiracetam, thyroid function, valproic acid

## Abstract

Background

Epilepsy, chronic monotherapy of antiseizure medications (ASMs), as well as the secondary disturbance of the thyroid axis, which includes both subclinical and clinical hypo- and hyperthyroidism, are crucial endocrine challenges during children’s development. In this study, the levels of thyroid hormones in children with epilepsy who underwent chronic monotherapy were compared with those of healthy children with no history of seizures and no experience with anticonvulsants. Moreover, in this study, the goal was to identify the actual rates, dynamics, and dose-dependence of thyroid dysfunction in the aforementioned study population.

Methodology

A prospective, observational, case-control study was conducted involving 150 patients with epilepsy on ASM treatment (cases) and 150 healthy control subjects who were matched for age and sex from a pediatric outpatient/inpatient department. The history, classification of seizures, and ASMs were documented. Serum thyroid-stimulating hormone (TSH), free triiodothyronine (fT3), and free thyroxine (fT4) were measured using the electro-chemiluminescent immunoassay test. Statistical analysis was performed using SPSS version 28 through one-way analysis of variance and paired Student’s t-test.

Results

Patients exhibited a significantly higher mean TSH level (3.82 ± 2.21 vs. 2.89 ± 1.12 mIU/L; p < 0.001) and a significantly lower mean fT4 level (1.28 ± 0.29 vs. 1.35 ± 0.31 ng/dL; p = 0.012), while fT3 characteristics did not change (p = 0.45). Analyses of subgroups revealed that atypical febrile seizures (4.56 ± 2.67 mIU/L) and generalized tonic-clonic seizures (4.12 ± 2.45 mIU/L) exhibited the most significant predisposition to extreme elevations in TSH levels (p = 0.004). Regarding treatment options, patients undergoing sodium valproate monotherapy presented the highest mean TSH levels (4.02 ± 2.11 mIU/L; p = 0.001), but levetiracetam appeared to be an effective thyroid-preserving choice and provided a more favorable fT4 profile (1.35 ± 0.33 ng/dL; p = 0.020). Regarding timing, the TSH peak occurred significantly between six and nine months of the drug administration period (4.35 ± 2.41 mIU/L; p = 0.024). In addition, a clear dose-dependent pattern was detected, with mean TSH levels rising progressively to a maximum of 5.20 ± 3.12 mIU/L within the highest 25-30 mg/kg dosing range (p = 0.008), representing a notable 31% endocrine elevation.

Conclusions

Children with epilepsy receiving maintenance therapies with sodium valproate or phenytoin have a considerable chance of suffering from subclinical hypothyroidism, which increases sharply between six and nine months of treatment at high doses. Thyroid function monitoring is strongly advised within the first year of treatment, along with dose adjustment and evaluation of thyroid-sparing drugs, such as levetiracetam.

## Introduction

Epilepsy, characterized by recurrent unprovoked seizures occurring at least 24 hours apart, is one of the most common neurological disorders in children, with a global prevalence of 0.5%-1% in the pediatric population [1]. The diagnosis typically requires either two unprovoked seizures or compelling electroencephalography and clinical evidence of persistent seizure predisposition [2]. Antiseizure medications (ASMs), such as carbamazepine, sodium valproate, phenytoin, and phenobarbitone, are the cornerstones of long-term seizure management [3]. In developing countries, the prevalence of pediatric epilepsy can reach up to 10 cases per 1,000 children [4]. While ASMs are effective in controlling seizures, their prolonged use is associated with systemic adverse effects, including endocrine dysfunction, metabolic disturbances, and hepatotoxicity [5]. Among these, thyroid dysfunction has emerged as a critical concern because of the pivotal role of thyroid hormones in growth, brain development, and metabolic regulation in children [6].

Thyroid hormones (triiodothyronine (T3) and thyroxine (T4)) and thyroid-stimulating hormone (TSH) are essential for neurodevelopment, particularly in young children. The hypothalamic-pituitary-thyroid axis tightly regulates their levels, but ASMs can disrupt this balance through hepatic enzyme induction, altered hormone metabolism, and direct effects on thyroid function [7]. Consequently, children on ASMs may develop subclinical or overt thyroid dysfunction, which can impair cognitive function, growth, and metabolic health [8]. Emerging evidence suggests that subclinical hypothyroidism, although often asymptomatic, may contribute to long-term developmental and metabolic complications [9]. Given these risks, routine thyroid monitoring in children receiving ASMs is increasingly recommended to enable early intervention and mitigate adverse outcomes [10]. Studies have indicated that enzyme-inducing ASMs (e.g., carbamazepine, phenobarbital, and phenytoin) accelerate thyroid hormone metabolism, reducing serum T4 and elevating TSH levels [11]. Conversely, valproic acid, a non-enzyme-inducing ASM, is linked to subclinical hypothyroidism with elevated TSH [12]. The prevalence of thyroid dysfunction varies by ASM type, dose, and treatment duration; for example, valproate is associated with a 15.2% risk of subclinical hypothyroidism after one year compared to 2.9% for phenobarbital [13]. Despite these findings, inconsistencies persist regarding the clinical significance of ASM-induced thyroid dysfunction, necessitating further research to clarify its long-term implications.

## Materials and methods

A one-to-one matched case-control design was utilized with a target enrollment of 150 cases and 150 age- and sex-matched controls (N = 300). From January 2024 to September 2025, 150 children (75 female and 75 male) in the age group of one year to 16 years who were diagnosed with epilepsy and were on ASM monotherapy (e.g., valproate, carbamazepine, levetiracetam) were enrolled from the general pediatric outpatient department for study and analysis. Another 150 children (75 male and 75 female) without epilepsy or thyroid disorders were selected from the same age group and recruited as healthy controls.

Sample size calculations were performed based on Dupont’s method [14] for matched pairs using Epi Info software [15]. Assuming a type I error rate (alpha) of 0.05 (two-tailed) and a statistical power (1-beta) of 0.80, a sample size of 150 matched pairs was sufficient to detect a minimum odds ratio of 2.20, assuming an exposure prevalence of 15% among the control population and a conservative matching correlation coefficient of 0.20. This sample size provided adequate statistical precision to evaluate the independent effects of antiseizure use on pediatric thyroid function profiles. Epilepsy was defined and classified using the International League Against Epilepsy guidelines [16].

After obtaining written informed consent from the parents/guardians of the children under study, clinical evaluation, history (seizure type, ASM duration, compliance), physical examination (goiter, hypothyroid symptoms), biochemical analysis (2 mL venous blood collected under aseptic conditions), and electro-chemiluminescent immune assay (ECLIA) testing (TSH, free T3 (fT3), free T4 (fT4) levels compared to age-specific norms) were recorded. ECLIA was performed using i-1000 (Abbott, Singapore) [17].

Data were collected and stored on a structured proforma (demographics, clinical/laboratory data). Statistical analysis was performed using SPSS version 28 (IBM Corp., Armonk, NY, USA). Data were analyzed using one-way analysis of variance (ANOVA) and paired Student’s t-test.

## Results

A total of 300 children were enrolled in the study. In the case group, 150 children with epilepsy were receiving ASM monotherapy, with 75 (50%) males and 75 (50%) females. The control group comprised 150 healthy children, of whom 75 (50.0%) were male, and 75 (50.0%) were female. Both groups of patients were aged between 1 and 16 years. No subjects were withdrawn or excluded from the study for any reason.

To test demographic matching within the study groups, a two-way ANOVA was performed using age as the dependent variable and group (case vs. control) and sex (male vs. female) as between-subject independent variables. There were no significant effects associated with group (F(1, 296) = 1.37, p = 0.243), or with sex (F(1, 296) = 0.30, p = 0.586). Importantly, the group-by-sex interaction was not statistically significant (F(1, 296) = 0.92, p = 0.338). This result proves mathematically that the groups were successfully age- and sex-matched (Table 1).

**Table 1:** Distribution of age and sex in the study population. Test applied: two-way analysis of variance. Statistical significance: P-value <0.05 and F-calculated value > F-critical value. SD = standard deviation; df = degree of freedom

Group	Sex	n	Mean age (years)	SD (years)	Source of verification	P-value	df-1, df-2	F-calculated value	F-critical value
Case	Male	75	7.45	4.21	Group (case vs. control)	0.243	1, 296	1.37	3.873
	Female	75	7.40	4.64	Sex (male vs. female)	0.586	1, 296	0.3	3.873
Control	Male	75	7.35	4.21	Interaction (group × sex)	0.338	1, 296	0.92	3.873
	Female	75	7.33	4.19	-	-	-	-	-

The study population exhibited diverse epilepsy subtypes, with epileptic syndromes being the most prevalent (27.3%, n = 41), followed by atypical febrile seizures (26.0%, n = 39). Generalized tonic-clonic seizures accounted for 18.7% (n = 28), while complex partial seizures represented 16.0% (n = 24). The stratification of thyroid hormone parameters between various forms of epilepsy was characterized by very large statistical differences in the profiles of serum TSH levels. Patients diagnosed with atypical febrile seizures and generalized tonic-clonic seizures showed higher mean serum TSH values (4.56 ± 2.67 mIU/L and 4.12 ± 2.45 mIU/L, respectively; p = 0.004). These results indicate a high sensitivity of children suffering from these types of epileptic seizures to the development of subclinical hypothyroidism. At the same time, patients with absence seizures showed the most stable endocrine values, as their baseline TSH levels remained normal (3.21 ± 1.54 mIU/L). In contrast to TSH, there were no statistically significant differences in thyroid hormones among seizure types. The elevated TSH levels in specific subtypes underscore the need for targeted monitoring, particularly in patients on valproate or phenytoin regimens. These findings highlight the importance of individualized thyroid surveillance based on both seizure type and ASM selection in pediatric epilepsy management (Table 2).

**Table 2:** Association of serum thyroid function parameters with epilepsy subtypes. Test applied: one-way analysis of variance. Statistical significance: *P-value <0.05 and **F-calculated value > F-critical value. TSH = thyroid-stimulating hormone; T3 = triiodothyronine; T4 = thyroxine; df = degree of freedom

Types of epilepsy	n = 150	Mean TSH (mIU/L)	Mean free T3 (pg/mL)	Mean free T4 (ng/dL)
Generalized tonic-clonic	28	4.12 ± 2.45	3.98 ± 0.71	1.21 ± 0.31
Complex partial	24	3.78 ± 1.98	4.02 ± 0.68	1.29 ± 0.28
Atypical febrile	39	4.56 ± 2.67	3.87 ± 0.63	1.32 ± 0.33
Epileptic syndrome	41	3.45 ± 1.87	4.11 ± 0.75	1.25 ± 0.27
Absence seizures	18	3.21 ± 1.54	4.23 ± 0.80	1.34 ± 0.30
P-value	-	0.004*	0.216	0.152
F-calculated value df (4,145)	-	3.95**	1.38	1.55
F-critical value	-	F-critical value (4,145, α = 0.05) ≈ 2.43		

Drug-specific stratification revealed highly varied changes in thyroid parameters for each ASM treatment regimen. Serum TSH was the highest among children receiving monotherapy with sodium valproate, with a mean value of 4.02 ± 2.11 mIU/L (p = 0.001), thereby suggesting the possibility of subclinical hypothyroidism in patients taking this drug. An intermediate level was noted in the case of phenobarbitone, where TSH was increased by 3.98 ± 2.34 mIU/L. Levetiracetam had the most positive impact on the endocrine system, maintaining near-normal TSH values at 3.12 ± 1.67 mIU/L. Peripheral thyroid hormone production differed between patients. Serum fT4 levels were significantly elevated in the levetiracetam group, with a value of 1.35 ± 0.33 ng/dL (p = 0.020). In contrast, serum fT3 did not differ and maintained a normal concentration across all treatment groups (p = 0.348) (Table 3).

**Table 3:** Association of serum thyroid function parameters with antiseizure medication types. Test applied: one-way analysis of variance. Statistical significance: *P-value <0.05 and **F-calculated value > F-critical value. TSH = thyroid-stimulating hormone; T3 = triiodothyronine; T4 = thyroxine; df = degree of freedom

Drug	n = 150	Mean TSH (mIU/L)	Mean free T3 (pg/mL)	Mean free T4 (ng/dL)
Sodium valproate	62	4.02 ± 2.11	3.89 ± 0.72	1.26 ± 0.29
Phenytoin	48	3.45 ± 1.89	4.02 ± 0.68	1.30 ± 0.31
Phenobarbitone	25	3.98 ± 2.34	3.95 ± 0.65	1.22 ± 0.27
Levetiracetam	15	3.12 ± 1.67	4.15 ± 0.80	1.35 ± 0.33
P-value	-	0.001*	0.348	0.020*
F-calculated value df (3,146)	-	5.24**	1.12	3.29**
F-critical value	-	F-critical value (3,146, α = 0.05) ≈ 2.67		

Time-based sub-analyses showed the presence of statistically significant temporal variations in the serum levels of TSH parameters depending on the varying periods of administration of the antiepileptic drugs. TSH levels were the highest during the early stages of ASM use, particularly at six to nine months, averaging 4.35 ± 2.41 mIU/L (p = 0.024), implying that this relatively short period may be regarded as a stage of high acute susceptibility to ASM-related thyroid changes. Beyond this stage, there appeared to be a degree of stabilization of TSH levels at nine to 12 months (3.67 ± 1.87 mIU/L) and long-term use (over 18 months of treatment) (3.45 ± 1.54 mIU/L). During the same period, there were no statistically significant variations in the peripheral thyroid hormones (Table 4).

**Table 4:** Association of serum thyroid function parameters with duration of therapy. Test applied: one-way analysis of variance. Statistical significance: *P-value <0.05 and **F-calculated value > F-critical value. TSH = thyroid-stimulating hormone; T3 = triiodothyronine; T4 = thyroxine; df = degree of freedom

Duration of therapy	n = 150	Mean TSH (mIU/L)	Mean free T3 (pg/mL)	Mean free T4 (ng/dL)
3–6 months	38	3.82 ± 1.98	4.02 ± 0.71	1.29 ± 0.31
6–9 months	42	4.35 ± 2.41	3.95 ± 0.68	1.25 ± 0.28
9–12 months	51	3.67 ± 1.87	3.98 ± 0.65	1.31 ± 0.30
12–18 months	15	3.91 ± 2.12	4.11 ± 0.80	1.23 ± 0.26
>18 months	4	3.45 ± 1.54	4.23 ± 0.82	1.28 ± 0.29
P-value	-	0.024*	0.482	0.109
F-calculated value df (4,145)	-	2.90**	0.86	1.90
F-critical value	-	F-critical value (4,145, α = 0.05) ≈ 2.43		

Therapeutic stratification of children under maintenance dosing showed that the 10-15 mg/kg level was the most common category, accounting for 27.3% (n = 41) of all pediatric cases. A low-level maintenance dose of 5 mg/kg was used in 12.0% (n = 18) of the subjects, while intermediate levels were administered in 21.3% (n = 32) of the subjects. High-level ASM doses above 20 mg/kg were used only in 20.7% (n = 31) of the cases. Statistical analysis demonstrated a clear dose-dependent association between increasing ASM requirements and thyroid parameters. The mean serum TSH level increased with increasing dosage from 3.98 ± 2.12 mIU/L in the lowest dose (5 mg/kg) category to 5.20 ± 3.12 mIU/L in the highest dose (25-30 mg/kg) category (p = 0.008). There was an increase of up to 31% in the mean TSH values in the highest operating dose categories. In parallel with increasing TSH, peripheral thyroid hormone levels did not show any statistical significance. While T3 showed a non-significant reduction from 3.92 pg/mL to 3.65 pg/mL (p = 0.42), T4 levels did not change at any tracking level (p = 0.61) (Table 5).

**Table 5:** Antiseizure medication dosage distribution and associated thyroid function parameters (n = 150). Test applied: one-way analysis of variance. Statistical significance: *P-value <0.05 and **F-calculated value > F-critical value. TSH = thyroid-stimulating hormone; T3 = triiodothyronine; T4 = thyroxine; df = degree of freedom

Dosage range (mg/kg)	n (%)	TSH (mIU/mL)	Free T3 (pg/mL)	Free T4 (ng/dL)
5 mg/kg	18 (12%)	3.98 ± 2.12	3.92 ± 0.68	1.31 ± 0.29
5–10 mg/kg	32 (21.3%)	3.75 ± 2.45	3.85 ± 0.71	1.29 ± 0.33
10–15 mg/kg	41 (27.3%)	3.82 ± 2.30	3.78 ± 0.67	1.30 ± 0.35
15–20 mg/kg	28 (18.7%)	4.10 ± 2.75	3.74 ± 0.73	1.27 ± 0.37
20–25 mg/kg	19 (12.7%)	4.55 ± 2.88	3.69 ± 0.76	1.24 ± 0.40
25–30 mg/kg	12 (8%)	5.20 ± 3.12	3.65 ± 0.80	1.20 ± 0.42
P-value	-	0.008*	0.42	0.61
F-calculated value df (5,144)	-	3.30**	0.97	0.77
F-critical value	-	F-critical value (5,144, α = 0.05) ≈ 2.28		

A comparison of biochemical studies between patients with epilepsy on ASM monotherapy and controls clearly showed differences in hormone levels. The TSH levels in the cases were significantly higher than those in the healthy group (3.82 ± 2.21 mIU/L vs. 2.89 ± 1.12 mIU/L; p < 0.001; t = 5.68). Compared with the control group, a significant decrease in the level of peripheral thyroid hormones was found in the study group based on the measurement of fT4 (1.28 ± 0.29 ng/dL vs. 1.35 ± 0.31 ng/dL; p = 0.012; t = -2.50). Unlike the above differences, the level of serum fT3 in both groups had similar configurations and did not differ (3.87 ± 0.67 pg/mL in cases vs. 3.92 ± 0.82 pg/mL in controls; p = 0.45; t = -0.70) (Table 6).

**Table 6:** Comparison of serum thyroid function parameters between epilepsy cases and healthy controls (N = 150 per group). Test applied: Student’s independent two-sample t-test (df = 296). Statistical significance: *P-value <0.05 and **t-value >1.96. TSH = thyroid-stimulating hormone; T3 = triiodothyronine; T4 = thyroxine; SD = standard deviation; df = degree of freedom

Tests	Group	Mean	SD	P-value	Calculated t-value
TSH (mIU/L)	Cases	3.82	2.21	<0.001*	t ≈ 5.68**
	Control	2.89	1.12		
Free T3 (pg/mL)	Cases	3.87	0.67	0.45	t ≈ -0.71
	Control	3.92	0.82		
Free T4 (ng/dL)	Cases	1.28	0.29	0.012*	t ≈ -2.50
	Control	1.35	0.31		

## Discussion

The present study provides a comprehensive evaluation of thyroid dysfunction in pediatric epilepsy patients receiving ASM therapy with significant implications for clinical practice. Cases (epileptic children on ASMs) and controls (healthy children) were matched successfully, minimizing confounding variables. This matching strengthened the internal validity of the present findings, ensuring that the observed differences in thyroid function were attributable to ASM exposure rather than demographic disparities as reported earlier [1]. Several studies have similarly emphasized the importance of age and sex matching in pediatric endocrine research, particularly given the dynamic changes in thyroid hormone levels during growth and development [18]. This alignment is crucial as thyroid function naturally varies with age, and even slight mismatches can distort results. The study exhibited a diverse distribution of epilepsy subtypes, with epileptic syndromes and atypical febrile seizures being the most prevalent. This heterogeneity reflects real-world clinical scenarios in which pediatric epilepsy encompasses a spectrum of seizure disorders with varying pathophysiologies. Notably, children with atypical febrile and generalized tonic-clonic seizures demonstrated the highest TSH levels, suggesting a subtype-specific susceptibility to ASM-induced thyroid dysfunction [19-22]. Mechanistically, this could be due to neuronal excitability differences influencing central thyroid regulation; ASM selection bias, where clinicians may preferentially prescribe certain drugs (e.g., valproate) for these subtypes; ASM prescription patterns and thyroid impact; and sodium valproate and phenytoin, which were the most frequently prescribed ASMs consistent with global trends in pediatric epilepsy management. Nevertheless, their strong association with elevated TSH levels underscores the critical trade-off between seizure control and endocrine side effects.

The mechanisms of ASM-induced thyroid dysfunction have been well reported, as valproate disrupts hepatic thyroid hormone metabolism, increasing TSH levels via reduced T4 to T3 conversion. Phenytoin induces hepatic enzymes (e.g., UDP-glucuronosyltransferases), accelerating thyroid hormone clearance [23], and levetiracetam exhibits a neutral thyroid profile, making it a preferable option for patients at risk of hypothyroidism [24]. These findings advocate for personalized ASM selection, particularly in children with preexisting thyroid vulnerabilities.

The temporal and dosage-dependent effects on thyroid function and the time-course of thyroid dysfunction revealed that the peak TSH at 6-12 months was an early critical window for thyroid disruption, whereas normalization after 12-18 months was possibly due to adaptive hormonal feedback mechanisms, as reported earlier [25]. This temporal pattern highlights the need for intensified thyroid monitoring during the first year of therapy, followed by gradual tapering. Likewise, the dose-response relationship revealed a clear dose-dependent rise in TSH, with levels increasing by 31% at the highest doses, i.e., 25-30 mg/kg, aligning with pharmacokinetic principles, where higher ASM doses exacerbate metabolic interference with thyroid function.

Limitations

While this study provides robust data regarding the impact of ASM monotherapy on pediatric thyroid function, a few limitations should be considered. Serum thyroid hormone levels were determined at one time point per patient during the corresponding therapy phase. There was no longitudinal tracking of serum hormone concentrations in the same child over time, beginning from baseline and extending over more than 18 months of uninterrupted treatment. There were no biochemical baselines before the administration of ASMs in the case group. Therefore, it was difficult to completely rule out the existence of any subclinical abnormality of the thyroid axis in some children before the administration of ASMs. Being a single-center study, the total number of participants (N = 300) was statistically sufficient; nevertheless, the stratification into subgroups generated relatively smaller numbers for certain parameters, such as in the subanalysis based on users of levetiracetam (n = 15) and that based on patients under medication for more than 18 months (n = 4). There was a lack of other diagnostic tests, including anti-thyroid peroxidase antibodies and thyroid ultrasound, which could distinguish between medication-related thyroid gland hypometabolism and autoimmune thyroiditis of either clinical or subclinical significance.

## Conclusions

The use of ASMs in children with epilepsy leads to profound changes in the hypothalamic-pituitary-thyroid axis, as exemplified by the significant elevation in TSH levels and decline in fT4 levels when compared to the control group. The described alteration in endocrine functions follows a particular clinical and dosage-dependent pattern. The peak of endocrine disruption was recorded between six and nine months after ASM administration, and the maximum level was achieved at high doses of 25-30 mg/kg/day. In addition, clinical characteristics such as individual drug choice played a crucial role in limiting this side effect. Thus, the application of valproate alone represents the most dangerous choice of ASMs, as it predisposes to subclinical changes in thyroid functions, while levotiracetam is an efficient substitute, maintaining normal fT4 levels. Moreover, the atypical febrile seizures and generalized tonic-clonic seizures represented the clinical type that exhibited the greatest susceptibility to increased levels of TSH. Therefore, regular testing for abnormalities in thyroid hormones should be performed in all pediatric patients with epilepsy under ASMs, especially after six to nine months from their introduction, focusing particularly on high-dose valproate.
